# Rotavirus Seasonality and Age Effects in a Birth Cohort Study of Southern India

**DOI:** 10.1371/journal.pone.0071616

**Published:** 2013-08-16

**Authors:** Rajiv Sarkar, Gagandeep Kang, Elena N. Naumova

**Affiliations:** 1 Department of Gastrointestinal Sciences, Christian Medical College, Vellore, TN, India; 2 Department of Civil and Environmental Engineering Tufts University School of Engineering, Boston, Massachusetts, United States of America; Columbia University, United States of America

## Abstract

**Introduction:**

Understanding the temporal patterns in disease occurrence is valuable for formulating effective disease preventive programs. Cohort studies present a unique opportunity to explore complex interactions associated with emergence of seasonal patterns of infectious diseases.

**Methods:**

We used data from 452 children participating in a birth cohort study to assess the seasonal patterns of rotavirus diarrhea by creating a weekly time series of rotavirus incidence and fitting a Poisson harmonic regression with biannual peaks. Age and cohort effects were adjusted for by including the weekly counts of number of children in the study and the median age of cohort in a given week. Weekly average temperature, humidity and an interaction term to reflect the joint effect of temperature and humidity were included to consider the effects of meteorological variables.

**Results:**

In the overall rotavirus time series, two significant peaks within a single year were observed – one in winter and the other in summer. The effect of age was found to be the most significant contributor for rotavirus incidence, showing a strong negative association. Seasonality remained a significant factor, even after adjusting for meteorological parameters, and the age and cohort effects.

**Conclusions:**

The methodology for assessing seasonality in cohort studies is not yet developed. This is the first attempt to explore seasonal patterns in a cohort study with a dynamic denominator and rapidly changing immune response on individual and group levels, and provides a highly promising approach for a better understanding of the seasonal patterns of infectious diseases, tracking emergence of pathogenic strains and evaluating the efficacy of intervention programs.

## Introduction

World-wide expansions of public health surveillance, long-term maintenance of patient electronic records and digital disease detection have invigorated attention to seasonal fluctuations in infectious diseases. A deep understanding of temporal patterns in disease occurrence and its governing principles is valuable for designing preventive programs for disease control, tracking effectiveness of public health programs, and allocating scarce resources.

Many infectious diseases exhibit seasonal patterns, when systematic periodic fluctuations are observed during an annual cycle [1], [2]. Seasonality can be characterized by the magnitude, timing, and duration of a seasonal increase. It may differ by pathogen and its strain virulence [3], [4], and may change from year to year due to shift/drift in antigenic strain and change in immunity of a naïve and exposed population [5]. Seasonal characteristics may also vary by population, geographical area, or climate zone [6], [7]. Introduction of a vaccine and/or successful coverage expansion [8], [9], [10] may also dramatically alter the seasonal fluctuations in infectious disease occurrence.

Periodic fluctuations can be successfully detected and modeled using statistical methods, which allows us to describe and compare seasonal characteristics: amplitude, peaks and troughs [11]. An analysis of seasonality has to be performed on a sufficiently long time series to be able to capture many occurrences of seasonal increases. Thus, by abstracting records from large databases of medical records, including laboratory tests, hospitalizations and mortality records, long time series can be successfully compiled to provide the foundation for reliable assessment of disease seasonality. However, each data source might contain only a fraction of all events associated with infection and sometimes may offer biased reflection of a true incidence of infection in a community.

While surveillance systems are an excellent source of data to study seasonality in a general population, they might not be ideal for tracking unique features of specific diseases. Cohort studies present a unique opportunity to explore intricate details associated with emergence of seasonal patterns. They allow longitudinal tracking of disease occurrence in each subject, to detect symptomatic and asymptomatic infection, to conduct complete genotyping and compare observed strains in the same individual, to link disease profile to environmental, social and genetic features.

At the same time, the specific features of cohort studies - staggered enrolment, age effect and short follow-up periods present substantial methodological challenges for seasonality analysis. Current analytical approaches for assessing seasonality are best suited for long time series, with relatively stable populations overall, and usually when the patterns of disease have a stable structure. It is useful to expand the methodology of seasonality analysis to this promising study design, which has already demonstrated advantages in understanding the natural history of viral and protozoan infections [12], [13], [14].

Rotavirus infection is a leading cause of morbidity in developing countries and currently there are a number of suitable vaccines. In temperate areas, seasonality of rotavirus infection with a single winter peak is well documented [15]. In tropical climates, its seasonality is less defined, however sometimes an increase is noticed during cold and dry time periods [7], [16]. Occasionally, in rotavirus two peaks within a single year are reported: one in winter and one in summer [17]. The burden of rotavirus diarrhea is highest among very young children and decrease rapidly thereafter [18], [19], [20], which can introduce a strong age-effect in the assessment of any temporal pattern.

In this paper, we assessed seasonal patterns of rotavirus in a birth cohort study of 452 children conducted in urban slums of Vellore in south India using specially developed models that account for staggered enrollment, age effect and environmental factors. We examined the joint effect of seasonality and age in three major genotypes of rotavirus, building on earlier published work where we demonstrated the epidemiology of rotavirus in a well-established birth cohort [13] and emphasized the importance of time-referenced covariates in quasi-experimental studies [21].

## Methods

### Ethics Statement

This paper presents the results of a secondary data analysis from a birth cohort study in southern India, which was approved by the Institutional Review Board of Christian Medical College, Vellore, India. Written informed consent obtained from parents or guardians of the participating children prior to enrollment also included permission to use the data for future research purposes. The time series presented in this study was assembled using de-identified data.

### Data Sources

We used data from a birth cohort study on the natural history of rotavirus infection in Vellore, south India [22], [23]. In this study, a total of 452 children were recruited between March 2002 and August 2003 and followed-up for a period of 3 years; a total of 373 (>80%) children completed the follow-up period. Each child was subjected to biweekly follow-up schedule to record diarrheal and other morbidities during the preceding days. Surveillance stool samples were collected every 2 weeks; and up to 3 diarrheal stool samples were collected every time the child had diarrhea. The stool samples were tested for the presence of rotavirus using ELISA and PCR based methods. The details of symptomatic and asymptomatic rotavirus infections over the 3-year period among the 373 children who completed the study have been reported elsewhere [13].

Data from all 452 children enrolled in this study was used to illustrate the seasonality of rotavirus diarrhea in this cohort. We defined a diarrheal episode to be associated with rotavirus if one or more stool samples collected within ±7 days of that episode was positive for rotavirus by PCR as has previously been described [24], [25]. Using this definition, we identified a total of 291 episodes of PCR-positive rotavirus diarrhea among the 452 children in this cohort. We also explored the effect of different rotavirus genotypes in the overall seasonality of rotavirus diarrhea among the study children.

Daily data on ambient temperature, relative humidity and rainfall for Vellore for the study period was obtained from the Regional Meteorological Office in Chennai, India. Average weekly temperature and humidity readings were then computed for the duration of the time series. The total volume of rainfall for a particular week was calculated using the daily rainfall measurements.

### Compilation of Time Series

eal event as the time stamp, or the time-referencing variable. All samples tested for rotavirus contained the date of sample collection; thus the created time series was 100% complete. Seasonality of rotavirus diarrhea was assessed by aggregating the daily surveillance data into a weekly time-series of counts. Diarrheal episodes were assigned to the week corresponding to the first day of that episode. A week was defined as seven consecutive days from Sunday to Saturday, irrespective of any overlap between two calendar years. The first week of January 2002 was considered to be the first week of the time-series. Hence, for this study, the week-series commenced from the 10^th^ week, beginning on March 10, 2002 and ended on the 243^rd^ week ending August 31, 2006, for a total of 234 weeks (see Dataset S1).

### Adjustment for Age and Cohort Effects

In cohort studies with staggered enrollment, there are at least three effects that might impact the time series: staggered enrollment, loss to follow-up and age effect. To address these factors, two additional time series were created: a time series of weekly counts of number of children (child-weeks of follow-up) in the study and a time series of median age of children in a given week (see text S1 for details). Using the weekly counts of children and events (rotavirus diarrhea), incidence rates per 1000 child-weeks of follow-up were calculated (see text S1 for details). Therefore, the assessment of seasonality was based on the weekly incidence rates, adjusting for the ageing in the cohort.

### Assessment of Seasonality

The seasonality of rotavirus diarrhea was assessed by fitting a Poisson harmonic regression (PHM) as a generalized linear model (GLM) considering two annual humps, which were accommodated by including four sine-cosine terms. To adjust for the cohort effect, we expanded the regression model by including child-weeks of follow up as a linear term. The age effect of rotavirus diarrhea was explored by applying three different models: first using a linear term representing the median age of the cohort for the corresponding week, second by adding a quadratic term for the weekly median age to the linear term and third by replacing the linear and quadratic terms by adding an exponential term representing the log-transformed median age of the cohort. Based on overall model performance and results interpretation, a linear term to represent the effect of age was selected. Finally, to consider the effects of meteorological variables, variables for weekly average temperature, humidity and an interaction term were included to reflect the joint effect of temperature and humidity. The sequentially-built final model can thus be depicted as below:
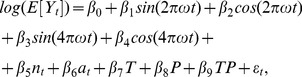
(1)where *t* is time in weeks, *ω* is frequency of 52.25, *Y_t_* is a time-series of incidence, *n_t_* is a term for weekly value of child-weeks of follow up; *a_t_* is a median age for a cohort at *t*-week, thus the regression parameter *β*_6_ reflect the change in the incidence associated with 1 week; *T*, *P* and *TP* are values for weekly average temperature, humidity and their cross-product interaction, respectively; *β* are regression parameters, *ε* is the error term. Using *β*_6_ we estimate the age effect as follow: 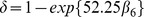, where 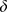 is the percent reduction in the incidence over one year. In this model, parameters *β*_1_ through *β*_4_ refer to seasonal harmonics. In fitting two sine-cosine harmonic pair we statistically tested that a pattern exhibit two peaks within a period of a full year and estimated the peak timing [26]. The quality of model fit was assessed as the difference between the null and regression deviance and expressed as percent (%) reduction in deviance. The sequential model building allows assessment of the contribution individual components in the total variability explained. The same model building structure was repeated for the three major rotavirus genotypes.

## Results

**Figure 1:**
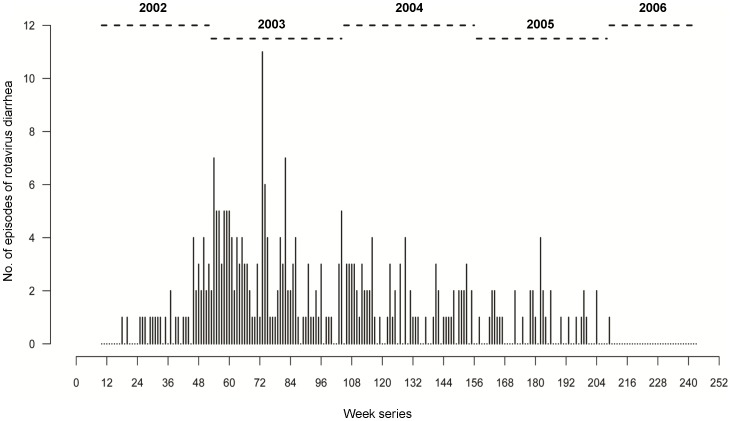
Needle plot showing the weekly distribution of counts of rotavirus diarrhea during the study period.

**Figure 2:**
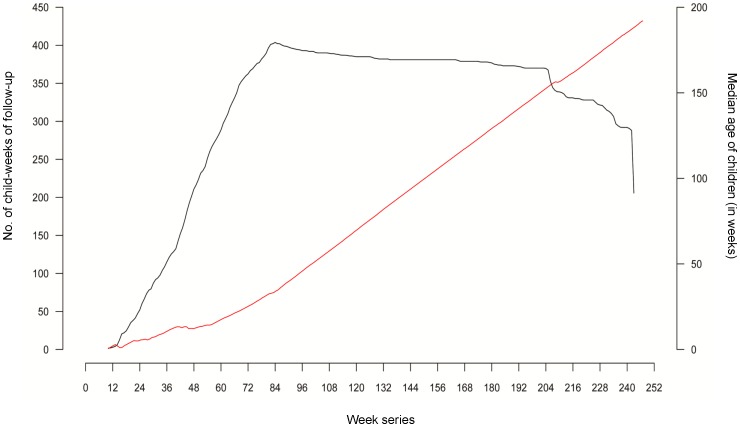
Cumulative weekly enrollment, follow-up and ageing of the cohort. The black line represents the total number of child-weeks of follow-up for each week in the time series. The red line depicts the median age of children (in weeks) corresponding to a particular week in the time-series.

**Figure 3:**
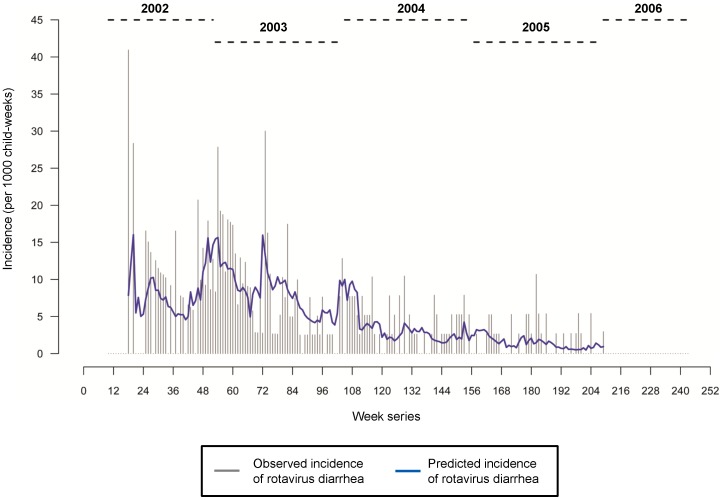
Week-series of incidence of rotavirus diarrhea (per 1000 child-weeks). The horizontal spikes depict the observed incidence of rotavirus diarrhea among children in the cohort. The blue vertical line depicts the predicted incidence derived from the Poisson harmonic regression model.

The weekly counts of detected rotavirus diarrhea (total 291 episodes) are plotted in a form of a needle-plot and presented in Figure 1. The time series (see Dataset S1) started from week 10, which refers to the beginning of enrollment. The observed average (95% CI) weekly incidence of rotavirus diarrhea was 4.4 (3.7–5.2) episodes per 1000 child-weeks. As expected, the initial weeks of the study had low counts of rotavirus diarrhea due to continuing enrollment. The highest count of rotavirus diarrhea was observed on the 73^rd^ week of the study, which coincides with the end of the enrollment period. The enrollment scheme is presented in Figure 2, which reflects three segments with characteristic features. Between 10^th^ and 84^th^ week a steady increase is observed in the child-weeks of follow-up depicting the ongoing enrollment process; between the 84^nd^ and 204^th^ week, a practically flat line in the child-weeks of follow-up was observed that denoted the time period when most of the children were being followed-up; thereafter, between 204^th^ and 243^rd^ a rapid decline was observed because of children completing the study. The ageing of the cohort is depicted in Figure 2 as a dashed line. After adjusting for staggered enrollment, the incidence rate revealed a steady decline from 40.9 and 28.3 episodes per 1000 child-weeks on 18^th^ and 20^th^ weeks respectively at the beginning to 2.9 episodes at 209^th^ week towards the end of the time series (Figure 3). This decline is likely to be associated with the age effect and the development of immune response.

In the overall rotavirus time series, two significant peaks within a single year were observed: one in winter and one in summer. Figure 3 reflects the predicted value of the regression model, which was built sequentially as described above. The parameters of the model and the goodness of fit are presented in Table 1. After adjustment for the age and cohort effect, the highest seasonal increase in rotavirus incidence was observed between the 41^st^ and 66^th^ weeks of the study, coinciding with the major bulk of rotavirus diarrhea counts among the study children. This wave peaked at 54^th^ week when a total of 7 episodes of rotavirus diarrhea were observed over 251.4 child-weeks of follow-up, resulting in an incidence of 27.8 episodes per 1000 child-weeks. The predicted incidence from the model was 15.7 episodes per 1000 child-weeks. The median age of the cohort was 14.2 weeks during this time.

**Table 1:** Results of the Poisson harmonic regression models for assessment of rotavirus seasonality, and age and cohort effects.

Effects		All rotavirus		G1P [8]		G2P [4]		G1P [4]	
	Model parameter	Value	*P*-value	Value	*P*-value	Value	*P*-value	Value	*P*-value
**Cohort effect**	Child-weeks of follow up	0.002	<0.001	0.003	<0.001	0.019	<0.001	0.002	0.005
**Age effect**	Median age of the cohort (in weeks)	−0.019	<0.001	−0.021	<0.001	−0.018	<0.001	−0.024	<0.001
**Seasonality effects**	Sine (1st harmonic)	−0.024	0.757	−0.692	<0.001	0.587	0.003	−0.220	0.371
	Cosine (1st harmonic)	0.328	0.006	0.757	0.019	−0.344	0.282	−0.701	0.04
	Sine (2nd harmonic)	0.277	<0.001	0.506	0.001	0.447	0.004	−0.052	0.746
	Cosine (2nd harmonic)	0.338	<0.001	−0.309	0.076	−0.491	0.013	0.738	0.001
**Effect of meteorological factors**	Weekly average temperature	0.821	<0.001	2.18	<0.001	0.341	0.383	0.282	0.447
	Weekly average humidity	0.308	<0.001	0.834	<0.001	0.234	0.158	0.192	0.212
	Temperature*humidity	−0.011	<0.001	−0.031	<0.001	−0.008	0.183	−0.005	0.341
	Null Deviance	1713	–	719	–	453	–	560	–
	Regression Deviance	959	–	527	–	288	–	398	–
	Percent reduction in deviance	43.9	–	26.7	–	36.5	–	28.9	–

Based on predicted curves, such seasonal increases were observed to repeat at least twice – one around week 72^nd^ and 84^th^ week and then another around 96^th^ and 108^th^ week. During the peaks of these intervals (81^st^ and 104^th^ week respectively), the predicted rotavirus incidence was found to be 9.9 and 9.1 episodes per 1000 child-weeks respectively; and the median age of the cohort was 32.9 and 55.3 weeks respectively. A smaller hump was also observed at the inception of the study, peaking at 25^th^ week when the first 68 children were enrolled. The median age of the cohort at this time was 6 weeks, thereby indicating neonatal and early childhood infections.

**Figure 4:**
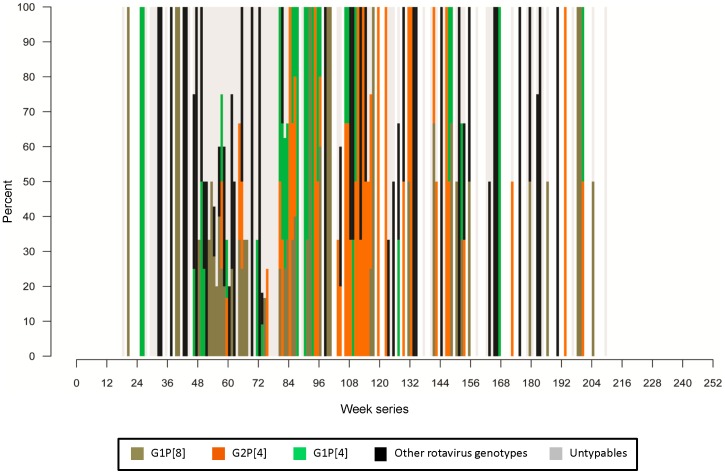
Weekly distribution of different rotavirus genotypes. Observed changes in a relative contribution of rotavirus genotypes indicate temporal clustering characteristic for seasonality in genotype dominance.

The cumulative weekly distribution and the relative contribution of the different rotavirus genotypes are presented in Figure 4. Visualization of these data suggests clustering of rotavirus genotypes at certain time points. For example, rotavirus G1P [8] was the predominant circulating genotype between the 52^nd^ and 67^th^ weeks, whereas between the 103^rd^ and 116^th^ week, G2P [4] was found to be more common. As observed in Figure 3, the second peak of rotavirus diarrhea seems to be driven primarily by the G1P [8] genotype, whereas the fourth peak was mostly driven by G2P [4]. Interestingly, the third peak observed in Figure 3 (81^st^ week) seems to be driven at least partially by G1P [4] and the untypable or partially typable rotavirus genotypes.

**Figure 5:**
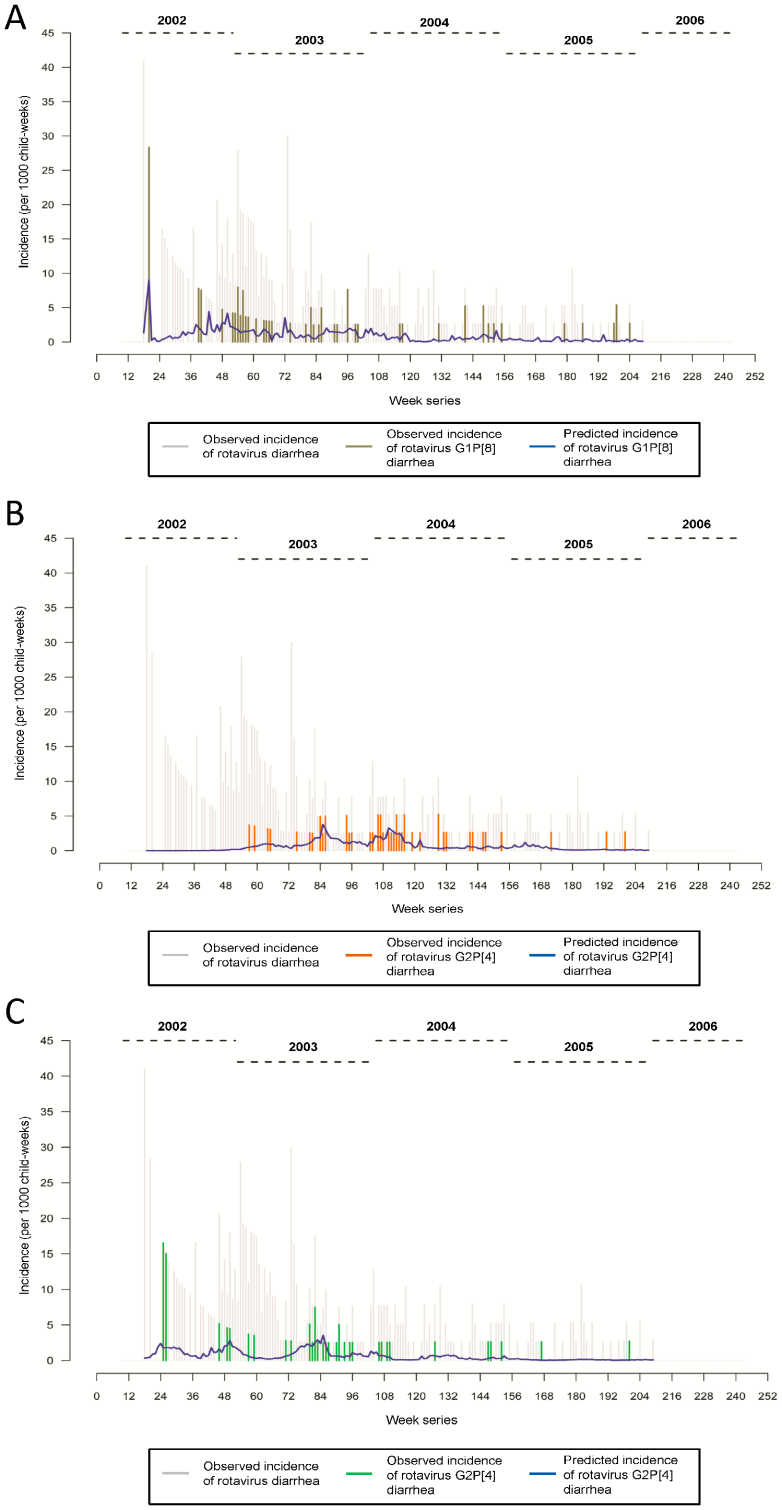
Week-series of incidence of diarrhea (per 1000 child-weeks) due to major rotavirus strains (A) G1P [8], (B) G2P [4], (C) G1P [4]. The colored horizontal spikes (khaki for G1P [8], orange for G2P [4] and green for G1P [4]) represent the observed strain-specific incidence of rotavirus diarrhea among children in the cohort. The vertical blue line depicts the predicted incidence derived from the Poisson harmonic regression model.

The time series depicting the seasonality in incidence rates for the three most common rotavirus genotypes along with their predicted values obtained from the final model is presented in Figure 5 (A-C). The first depicted seasonal wave of the predominant rotavirus genotype G1P [8] (see Figure 5A) observed in this study coincides with the second seasonal increase for overall rotaviruses (see Figure 3). The most pronounced seasonal increase of G2P [8] (Figure 5B) coincides with the fourth overall rotavirus seasonal hump (see Figure 3). Similarly, the first three peaks of G1P [4] coincides with the first three increments in the overall incidence of rotavirus diarrhea, which suggest that the observed temporal clustering of rotavirus diarrhea can be driven by multiple strains, with predominant contributions from a single strain. For the overall rotavirus incidence and for the individual strains assessed, the seasonality remained significant after adjusting for meteorological parameters, and the age and cohort effects (Table 1).

Over the course of the study, the effect of age was found to be the most significant contributor for rotavirus incidence. In one year, a significant reduction of 62.9, 66.6, 60.9, 71.5 percent was observed in the incidence of rotavirus diarrhea and three major genotypes, G1P [8], G2P [4], and G1P [4], respectively (p<0.0001, see Table 1). The age-specific decline explained 32.1% of the variation in the incidence of rotavirus overall, whereas the seasonality and meteorological factors explained 6.1% of the overall model variation. The age effect varied from one strain to another. For rotavirus G1P [8], age effect contributed to only 9.8% of the variation, whereas seasonality and meteorological factors together explained 13.3%. Similarly, for G2P [4], the contribution for age-effect, and seasonality and meteorological factors was 12.1% and 6.4% respectively. Among the common rotavirus strains, the age effect was most prominent for G1P [4] accounting for 16.6% of the overall variation (see Figure 5C); the relative contribution of seasonality and meteorological parameters for this strain was 10.1%.

## Discussion

A time series was created for rotavirus diarrhea observed in a birth cohort study in southern India. We adjusted for age and cohort effects, and assessed the seasonal patterns of rotavirus diarrhea overall and that of three most common genotypes using a Poisson harmonic regression model. After adjusting for strong age effect, seasonal fluctuations remained significant with biannual peaks, 6 months apart. The most pronounced peak was observed during the first winter in the life of the newborn at an average age of about 4 months.

Birth cohort studies offer an unbiased approach to examine relationship between time of birth and susceptibility to infections and have substantial advantages in tracking unique features of specific diseases such as rotavirus where the incidence of symptomatic disease is heavily shifted towards the first few years of life and then diminishes rapidly. It is also allows careful tracking of age effects and changes in immunity, and frequency and routes of exposure due to changes during child development including feeding patterns, introduction of weaning foods and changes in hygiene practices.

A strong age effect was noticed in this study, with the incidence of rotavirus diarrhea decreasing steadily with increasing age. This is possibly due to the development of protective immunity to rotavirus, which prevented children from developing symptomatic infections at an older age. Natural rotavirus infection has been shown to confer protection against subsequent infection and disease [27], [28]. Despite this strong age effect, the detection of seasonal fluctuations in the incidence of rotavirus infection is, on its own, remarkable.

The temporal clustering of the circulating rotavirus genotypes, even within a relatively short time series (Figure 4) was another important finding of this study, and is possibly a result of immune selection pressure. Also, the strain-specific variations in the seasonality of rotavirus observed in this study suggest that the seasonality of rotavirus may be strain-dependent. Therefore, in future studies, including data on the circulating rotavirus genotypes may provide a more robust estimate of the seasonality of rotavirus infection.

It has previously been observed that in temperate climates, the season of birth can adversely affect rotavirus diarrhea and children born in summer have a higher risk of laboratory-confirmed rotavirus infection that typically peaked in winter [29]. However, unlike in temperate climates, in this study two seasonal peaks of rotavirus diarrhea, 6-months apart, were observed which suggests that children in tropical climates may have the same risk of exposure to the virus, irrespective of their birth season. The staggered enrollment lasted for over one year (Figure 2), thereby enabling children born at different seasons to be equally represented in this study. Hence, it can reasonably be assumed that the effect of seasonality observed in this study was independent of their birth season.

**Figure 6:**
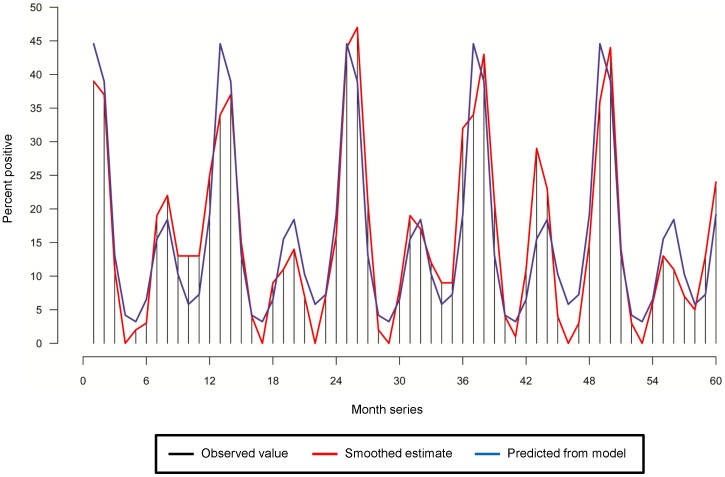
Seasonality of rotavirus diarrhea in Dhaka, Bangladesh (2001–2006) with pronounced biannual peaks. The horizontal spikes depict the observed monthly values, the red vertical lines represent the smoothed estimates, and the blue vertical lines reflect the predicted counts based on Poisson harmonic regression model with four sine-cosine terms.

Cohort studies with an intensive follow-up are difficult to replicate and such in-depth evaluation can be available only for a limited time period, small geographic area and relatively small sample size. To increase the power to appropriately detect and characterize seasonality it would be valuable to compile data from similar studies, and to pair these data with ongoing community and hospital based surveillance. The observation of biannual peak agrees with other studies of rotavirus in tropical climates [17], [30], [31], [32], [33]. Published data also provide an illustration of biannual peaks [17]. In a cross-sectional study in Dhaka, a total of 10,739 stool samples from patients admitted between January 2001 to May 2006 in the ICDDR,B hospital were tested for the presence of rotavirus using ELISA-based methods, of which 2706 (25.2%) were found to be positive. A large proportion (>90%) of the rotavirus positive patients were children below the age of 2 years. Applying the harmonic regression model presented in this study, a predictive curve was produced that accounted for 80% of variability in the monthly time series of the proportion of rotavirus positive stool samples (see Figure 6). Although the shift to more severe cases due to difference in patient profiles has to be taken into account while comparing cohort data with community or hospital based surveillance systems [24], this similarity of biannual peaks observed in the large surveillance sample and our relatively small cohort is encouraging.

The methodology for assessing seasonality in cohort studies is not yet developed and this study is the first attempt to explore seasonal patterns in a sample with a dynamic denominator and rapidly changing immune response on an individual and group levels. A significant seasonal fluctuation was detected in this tropical setting and moderate explanatory power was achieved with widely accepted harmonic regression models adapted to a carefully crafted weekly time series [11], [34], [35]. Examination of weekly rates substantially improves the evaluation of seasonal curves when compared to monthly data traditionally used in the vast majority of epidemiological studies, which limit the ability to detect significant differences in the seasonal peaks masked by cumulative aggregates. The specific features of cohort studies might trigger the development of a new generation of statistical models tailored to such designs and foster the understanding of temporal variations in disease incidence.

There is paucity of data from prospective, community-based studies that measure the incidence of rotavirus diarrhea in the Indian children. The majority of studies on rotavirus diarrhea are hospital-based cross-sectional studies. From these studies, the prevalence of rotavirus among hospitalized children was found to range from 19.2–49.9% (average 33.6%). The proportion of diarrhea attributed to rotavirus was, however, much lower at 12% (range 7.1–15.5%) in outpatient settings [36]. Converting our observed weekly incidence of 4.4 (3.7–5.2) episodes per 1000 child-weeks to an annual incidence would have resulted in an average (95% CI) of 0.22 (0.19–0.27) episodes of rotavirus diarrhea per child per year. This is comparable to the rotavirus diarrheal incidence of 0.16 (0.10–0.22) episodes per child-year among <2-year old children in an urban slum in Delhi [37].

While birth cohort studies provide highly valuable information to close critical gaps in understanding disease burden, the design of such studies should be also explored as a working platform for investigating the emergence of novel pathogens and strains. In this study, a large number of untypable samples occurred before the switch to another type (Figure 3). Their disproportional increase might indicate appearance of a new strain previously unknown and not seen for a period of time and not considered for typing. Using a wide range of information sources through triangulation and integration of epidemiological cohorts, surveillance systems, hospitalization records and outpatient follow-up is a highly promising approach for a better understanding of the seasonal patterns of infectious diseases, tracking emergence of pathogenic strains and evaluation the efficacy of intervention programs.

## Supporting Information

Dataset S1 Time series of selected variables presented in the study.
(XLS)

Text S1 Adjustment for age and cohort effects.
(DOC)
