# Correction: Rotavirus Seasonality and Age Effects in a Birth Cohort Study of Southern India

**DOI:** 10.1371/annotation/2f5b6cf8-926b-4397-9a89-64544cc7d512

**Published:** 2014-01-16

**Authors:** Rajiv Sarkar, Gagandeep Kang, Elena N. Naumova

All instances of the names of the three major rotavirus strains, G1P[8], G2P[4] and G1P[4] are incorrectly marked with references. The number in brackets should not be separate from the rest of the rotavirus strain, and refers to the rotavirus P type, not a reference number.

In the "Compilation of Time Series" under the methods section, a part of the first sentence is missing. The first sentence should read, "To create a time series of counts, we used the date corresponding to the first day of the diarrheal event as the time stamp or the time referencing variable."

