# Supplementary material for: Rotavirus Seasonality and Age Effects in a Birth Cohort Study of Southern India
Source: PLoS One. 2013 Aug 16;8(8):e71616. doi: 10.1371/journal.pone.0071616 (PMC3745434; doi:10.1371/journal.pone.0071616)
Supplement: Text S1 — Adjustment for age and cohort effects. (DOC) [file pone.0071616.s002.doc]

**Calculation of the parameters used for adjustment of age and cohort effects**

**Adjustment for staggered enrollment and loss-to-follow-up:**

Staggered enrollment and loss-to-follow up was adjusted for by computing a time series of counts of number of children followed-up at each week during the study period. The compilation of the time series is depicted in the using the following flow-diagram:

Compute a daily time-series for each child using the dates of commencement and end of follow-up

Add the daily time series of each child to calculate the total number of children followed-up for each day of the study (child-days of follow-up)

Calculate the total number of child-days of follow-up for each week by adding the total number of child-days of follow-up for that week

Divide the total number of child-days of follow-up for each week by 7 to calculate the total number of child-weeks of follow-up for the corresponding week

**Adjustment for age-effect:**

In order to adjust for the age-effect, a time series of median age of children for each week of the study was calculated using the following notation:

Median age of cohort in week *i* (*M_i_*) = median (*A_ji_*), where *A* is the age of *j*-th child in the *i*-th week.

**Obtaining the weekly incidence rates of rotavirus diarrhea:**

The incidence rates per 1000 child-weeks of follow-up were calculated using the following method:

Let *N_i_* be the weekly number of children under observation for *i*-th week and *C_i_* be the weekly counts of rotavirus diarrhea. Then, the weekly incidence (per 1000 child-weeks) of rotavirus diarrhea (*R_i_*) can be depicted as:

*R_i_* = (*C_i_* / *N_i_*) × 1000
